# Barley (*Hordeum vulgare*) in the Okhotsk culture (5th–10th century AD) of northern Japan and the role of cultivated plants in hunter–gatherer economies

**DOI:** 10.1371/journal.pone.0174397

**Published:** 2017-03-29

**Authors:** Christian Leipe, Elena A. Sergusheva, Stefanie Müller, Robert N. Spengler, Tomasz Goslar, Hirofumi Kato, Mayke Wagner, Andrzej W. Weber, Pavel E. Tarasov

**Affiliations:** 1 Institute of Geological Sciences, Section Paleontology, Freie Universität Berlin, Malteserstr. 74–100, Building D, Berlin, Germany; 2 Institute of History, Archaeology and Ethnography, Far Eastern Branch of the Russian Academy of Sciences, Pushkinskaya 89, Vladivostok, Russia; 3 Eurasia Department and Beijing Branch Office, German Archaeological Institute, Im Dol 2–6, Building II, Berlin, Germany; 4 Institute for the Study of the Ancient World, New York University, New York, NY, United States of America; 5 Max Planck Institute for the Science of Human History, Jena, Germany; 6 Faculty of Physics, Adam Mickiewicz University, Umultowska 85, Poznan, Poland; 7 Poznan Radiocarbon Laboratory, Foundation of the A. Mickiewicz University, Rubiez 46, Poznan, Poland; 8 Center for Ainu and Indigenous Studies, Hokkaido University, Kita 8, Nishi 6, Kita-ku Sapporo, Hokkaido, Japan; 9 Department of Anthropology, University of Alberta, Tory Bldg. 13–15, Edmonton, Alberta, Canada; 10 Aix Marseille Univ, CNRS, Minist Culture & Com, LAMPEA, Aix-en-Provence, France; New York State Museum, UNITED STATES

## Abstract

This paper discusses archaeobotanical remains of naked barley recovered from the Okhotsk cultural layers of the Hamanaka 2 archaeological site on Rebun Island, northern Japan. Calibrated ages (68% confidence interval) of the directly dated barley remains suggest that the crop was used at the site ca. 440–890 cal yr AD. Together with the finds from the Oumu site (north-eastern Hokkaido Island), the recovered seed assemblage marks the oldest well-documented evidence for the use of barley in the Hokkaido Region. The archaeobotanical data together with the results of a detailed pollen analysis of contemporaneous sediment layers from the bottom of nearby Lake Kushu point to low-level food production, including cultivation of barley and possible management of wild plants that complemented a wide range of foods derived from hunting, fishing, and gathering. This qualifies the people of the Okhotsk culture as one element of the long-term and spatially broader Holocene hunter–gatherer cultural complex (including also Jomon, Epi-Jomon, Satsumon, and Ainu cultures) of the Japanese archipelago, which may be placed somewhere between the traditionally accepted boundaries between foraging and agriculture. To our knowledge, the archaeobotanical assemblages from the Hokkaido Okhotsk culture sites highlight the north-eastern limit of prehistoric barley dispersal. Seed morphological characteristics identify two different barley phenotypes in the Hokkaido Region. One compact type (naked barley) associated with the Okhotsk culture and a less compact type (hulled barley) associated with Early–Middle Satsumon culture sites. This supports earlier suggestions that the “Satsumon type” barley was likely propagated by the expansion of the Yayoi culture via south-western Japan, while the “Okhotsk type” spread from the continental Russian Far East region, across the Sea of Japan. After the two phenotypes were independently introduced to Hokkaido, the boundary between both barley domains possibly existed ca. 600–1000 cal yr AD across the island region. Despite a large body of studies and numerous theoretical and conceptual debates, the question of how to differentiate between hunter–gatherer and farming economies persists reflecting the wide range of dynamic subsistence strategies used by humans through the Holocene. Our current study contributes to the ongoing discussion of this important issue.

## Introduction

Barley is the fourth most important cereal cultivated in the world today, after maize (*Zea mays* ssp. *mays*), rice (*Oryza sativa*), and free-threshing bread wheat (*Triticum aestivum*) [1]. Domesticated barely evolved under human selective pressure from a two-rowed, hulled, narrow-grained, and brittle-rachised wild form (*Hordeum vulgare* ssp. *spontaneum*). While there has been a longstanding debate over the origins and spread of barley, the currently accepted view suggest that it was morphologically domesticated multiple times, but that all of these domestication processes took place within the Fertile Crescent of Southwest Asia. The fixation of the tough-rachis mutation, the first phenotypical trait of domestication, into the cultivated barley population took several millennia and occurred in farming communities across the Crescent in parallel. As Willcox [2] and others have recently pointed out, there is roughly contemporaneous evidence for the gradual domestication of wheat and barley at several different sites spanning from the southern Levant to western Iran [3], dating to the early Pre-Pottery Neolithic, i.e. more or less simultaneous with the end of the last glacial period and the onset of the Holocene interglacial.

For a long time, scholars studying the economies of pre-industrial peoples around the world have discussed their subsistence economies in polarised terms, either clumping them into the category of hunter–gatherers or farmers. There is increasing awareness among academics that past societies do not always fit neatly into these categories and that there is an array of economic variation melding parts of each category. Besides foraging societies that are characterised by a long-term transformation process towards agriculture (e.g. the Mesolithic/Neolithic transition in Europe), many other peoples incorporated wild plants and/or small-scale food production of domesticated plants and/or animals into long-term sustainable subsistence strategies. Well-known examples include the Hopewell of eastern North America (e.g. [4]), the Pacific Northwest Coast societies (e.g. [5]), the Shoshonean groups of the North American Great Basin (e.g. [6]), groups of the Highlands of New Guinea (e.g. [7]), and the Jomon of Japan (e.g. [8]). Much research has been conducted that illustrates the great complexity and variability of subsistence strategies of groups that can be placed somewhere between hunting–gathering and agriculture (e.g. [9] and references therein). Despite a large body of studies and numerous theoretical and conceptual debates, the question about how to classify present and past “middle ground” societies persists reflecting a major problem in current archaeology and in understanding subsistence strategies.

Besides studying the timing and locations of crop domestication, two of the most important themes in the archaeobotanical sciences have been (1) how cereals spread into different regions of the world and (2) how prehistoric cultures switched to productive economies, i.e., moved from foraging to farming. Researchers are reconstructing the dispersal of barley and other crops of the Southwest Asian agricultural package out of their 'core area'. In addition to rapidly dominating the economy of peoples in Neolithic Europe, barley spread eastward across the Asian mainland. Barley arrived in the greater Indus valley region and southern Turkmenistan by ca. 6000 cal yr BC [10], to monsoon-dominated India after 3000 cal yr BC [10] and the mountain foothills of Kazakhstan in Central Asia by 2200 cal yr BC [11], to Central China [12], the Tibetan Plateau [13] and the Korean Peninsula [14] around 1500 cal yr BC, and to the Primor’e Region in the Russian Far East (RFE) after 400 cal yr BC [15].

**Fig 1:**
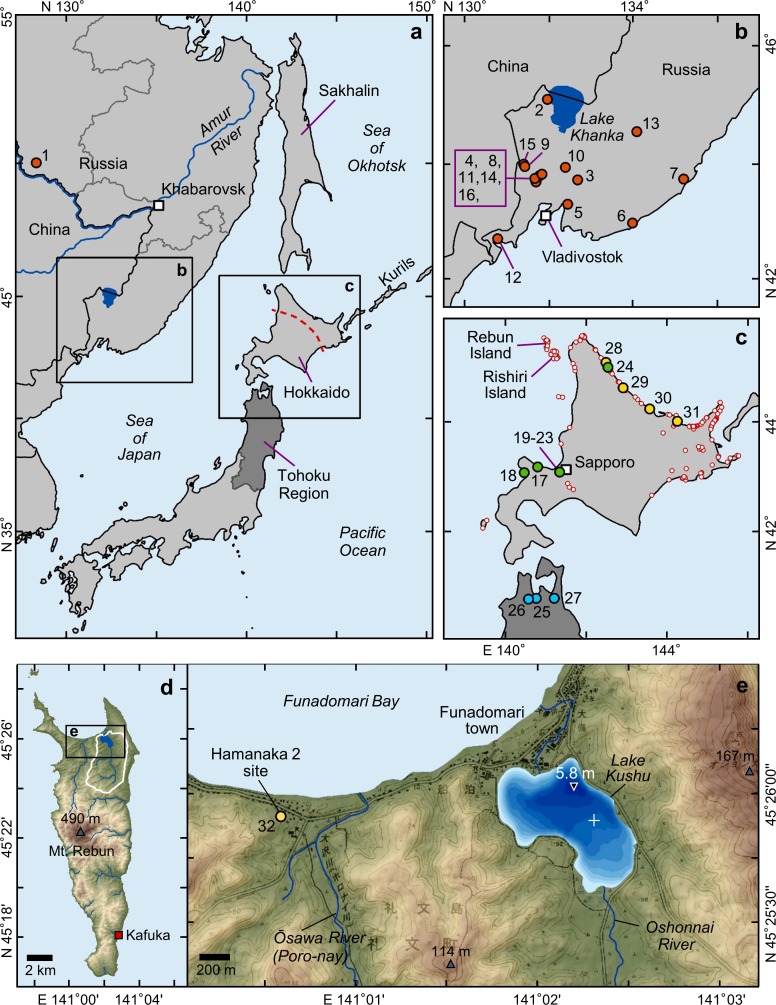
Location maps of the study region, Hamanaka 2, the RK12 coring site and other archaeobotanical records discussed in the text. Map compilation showing (A) the location of the study region in the northwest Pacific region; (B) the southern Primor’e Region (Russia); (C) the Hokkaido and northern Tohoku regions; (D) Rebun Island; and (E) the northern part of Rebun with Lake Kushu (white cross indicates location of the RK12 coring site) and the Hamanaka 2 archaeological site. Dots illustrate the locations of Okhotsk culture (yellow), Satsumon culture (green), Heian period (blue), and the RFE Iron Age–Eastern Xia State (red) barley records used for seed morphological comparison (see S3 Table for site names and further details). Red circles in (C) show the location of the 194 Okhotsk culture sites listed in the Hokkaido archaeological site database (http://www2.wagamachi-guide.com/hokkai_bunka/). The dashed line marks the proposed boundary of where the two barley morphotypes dominate. Bathymetry of Lake Kushu (0.5 m isolines) is based on survey data provided by T. Haraguchi (Osaka City University). Topographic maps are based on data from the elevation Shuttle Radar Topography Mission (SRTM) V4.1 [24]. Isolines for the terrestrial area in (E) are drawn from a topographic map [25].

The only archaeobotanical data we have for its spread into the northwest Pacific islands comes from the Japanese archipelago, with no studies thus far conducted in Sakhalin and the Kurils [16]. Japanese scholars claim that preserved barley grains have been sporadically recovered from archaeological excavations on Honshu and Kyushu islands (see [17] and references therein) from contexts dating to the Middle–Final Jomon period (ca. 3500–100 cal yr BC, according to [18]). However, intensive and widespread cultivation of barley, as well as rice and wheat does not appear to have been present prior to the Initial Yayoi period on Kyushu (ca. 1000–900 cal yr BC, see [18] and references therein) and around 100 cal yr BC (according to [18]) on north-eastern Honshu. Thus, the Jomon/Yayoi transition in central and southern Japan marks the switch from an economy dominated by foraging to one based on agricultural production. In contrast, the Hokkaido Region (Fig 1C) was not affected by these changes in subsistence. People in northern Japan, similar to those in Greenland, Arctic regions of Asia, and the American West Coast, remained “complex” hunter–fisher–gatherer well into the historic period [19]. Local Jomon populations of Hokkaido continued a foraging lifestyle [20] until the middle of the 1st millennium AD when they were replaced by Okhotsk cultural communities in the north and by Satsumon cultural communities in the central and the southern parts of the island [21]. Both of the latter cultures are commonly identified as hunter–fisher–gatherers [19]; however, their archaeological remains show evidence for the use of metals and the cultivation of crops [8]. The extent of their productive economy has not been fully studied. To date, the only clear evidence for the cultivation of barely and other crops by Satsumon people in the late 1st millennium AD comes from a single excavation site, which is located in the municipality of Sapporo [8]. There are more data showing that their contemporary neighbours to the north, the Okhotsk culture in Hokkaido, were cultivating both broomcorn (*Panicum miliaceum*) and foxtail (*Setaria italica*) millet and barley [8]. However, there have only been a few archaeobotanical studies on Okhotsk sites in Hokkaido and they are entirely published in local Japanese periodicals (e.g. [22, 23]), which are often not available to the international scientific community.

This study presents recently recovered barley grains that have been radiocarbon dated to the second half of the 1st millennium AD corresponding to the Okhotsk culture period in northern Hokkaido; so far they represent the north-eastern limit of barley dispersal in Eurasia before the modern era. The macrobotanical remains were recovered through water flotation of sediment samples from several excavation campaigns at the Hamanaka 2 archaeological site, Rebun Island (Fig 1), conducted by the international Baikal–Hokkaido Archaeology Project (BHAP: http://bhap.artsrn.ualberta.ca). The results of the macrobotanical analysis are discussed in the context of plant use and subsistence strategies. These well-preserved barley specimens from Rebun Island are accurately dated and morphometrically described. We also compiled a new morphological dataset of archaeological barley grains including the Hamanaka 2 record as well as published and unpublished records from other sites in the wider study region. The morphological dataset is further used to discuss dispersal pathways to extend the existing “map” of chronological and geographical spread of domesticated barley throughout East Asia. Additionally, a detailed pollen analysis of contemporaneous sediment layers from the bottom of Lake Kushu, located close to the Hamanaka 2 site (Fig 1) is employed in the current study in order to reconstruct changes in the local vegetation and to discuss human impacts on the island’s environment prior, during, and after the main Okhotsk culture occupation phase.

## The Okhotsk culture

The people of the Okhotsk archaeological culture are regarded as a hunter–gatherer society with an economy that strongly relied on marine resources. They occupied a widespread maritime environment, mainly along the southern and eastern littoral margins of the Sea of Okhotsk including northern and north-eastern Hokkaido (see Fig 1C for archaeological site distribution), Sakhalin Island, and the Kurils (Fig 1A). In the Hokkaido Region, the peak of the Okhotsk cultural occupation dates from the 6th to the 8th century AD (see [26] and references therein). Based on pottery style, the “Okhotsk cultural sequence” in northern Hokkaido is divided into three chronological stages comprising the (1) Susuya culture (2nd–5th century AD), which is often referred to as incipient or Proto-Okhotsk, (2) the Towada, Kokumon, Chinsenmon, Haritsukemon, and Somenmon cultures (6th–8th century AD) regarded as the main stages, and (3) the Motochi culture (9th–10th century AD) as the final stage [27]. While Okhotsk cultural traits persisted through the Tobinitai period in eastern Hokkaido until the 12th century AD, replacement or assimilation of the Okhotsk culture in northern Hokkaido by Satsumon/Proto-Ainu populations originating from the central and southern areas of Hokkaido was completed by the end of the 10th century AD [26].

Archaeologists believe that the Okhotsk culture people migrated to Hokkaido from the north (i.e. Sakhalin Island), first occupying Rebun and Rishiri islands as well as the northern tip of Hokkaido and subsequently dispersing eastwards along the coast of the Sea of Okhotsk [28]. Results of archaeological and genetic studies suggest that the Okhotsk population probably originated from the lower Amur River basin (e.g. [29–31]). The population spread onto the islands bordering the Sea of Okhotsk, which is believed to have been due to socio-political conflict [31]. There is also evidence for the onset of cooler climatic conditions in the lower Amur River basin around the end of the 1st millennium BC [32, 33]. These climate changes may have played a role in the southward spread (ca. 500 AD) of these people to Hokkaido [34] and their later absorption/replacement (by ca. 1000–1200 cal yr AD; [26, 27]).

A defining trait of the Okhotsk culture is its subsistence strategy, traditionally thought to be a specialised system of marine resource extraction [26, 35]. This is reflected by the geographic distribution of sites along coastal regions (Fig 1C) and confirmed by archaeological studies of faunal remains and tool assemblages, which indicate intensive marine hunting, fishing, and gathering activities (e.g. [31, 36, 37]). Nitrogen stable isotope studies on human remains also point to a diet with high proportions of protein derived from marine organisms (e.g. [35, 38, 39]). Analysis of human bone collagen revealed a relative contribution of marine protein in the range of 60–94% for individuals from Rebun Island [38] and 80–90% for individuals from eastern Hokkaido [39]. However, there is enough evidence to suggest that the diet of the Okhotsk people may have been much more diverse than the isotopic data imply. People likely supplemented the maritime resources with terrestrial mammals such as deer, fox, rabbit, and marten [37]. Cut marks on bones from domesticated dogs suggest that they were also part of the diet [36], and remains of domestic pigs are limited to northern Hokkaido [26]. In addition, there is evidence for the use of edible wild plants including *Aralia* (spikenard), *Polygonum* (knotweeds), *Actinidia* (Chinese gooseberry), *Vitis* (grapevines), *Sambucus* (elderberry), *Empetrum nigrum* (crowberry), *Rubus* sp. (blackberry), *Phellodendron amurense* (Amur corktree), and *Juglans* (walnut). Furthermore, as already noted, broomcorn millet, foxtail millet, and barley grains have been recovered from sites in this cultural horizon (see [26] and references therein). Admittedly, we know very little about the role of any of these plants in the economy, or whether the crops had a dietary or ritual function [22].

## Study site and environmental setting

The archaeobotanical samples presented in this paper were collected from the archaeological site of Hamanaka 2. This shell-midden site is located on the coast of the Funadomari Bay on the northern part of Rebun Island, which lays 45 km east of the northern tip of Hokkaido Island (Fig 1C–1E). For nearly a century, archaeologists have recognised the abundance of archaeological remains dating to the Okhotsk cultural period in the Funadomari Bay area [40]. Archaeological excavations started in the region in 1949, focusing on the Hamanaka 2 site complex and unearthed pottery, hearths, shell-middens, marine mammal remains, human burials, and house pits, suggesting residential activities at the site during the Okhotsk period [41]. The most recent excavation campaign at Hamanaka 2 was conducted by the BHAP starting in 2011. The site deposits constitute a well-stratified shell-midden on top of a sand-dune formation roughly 100 m south of the current coast line. So far, archaeological finds include human and dog burials, pig remains, ceramic and lithic artefacts, and abundant remains of sea mammals, fish, and shellfish spanning the occupation periods of the Late, Final, and Epi-Jomon as well as Satsumon, Okhotsk, and Ainu cultures [42] between the 2nd millennium BC and mid-19th century AD (see [43] and references therein).

The climate of Rebun Island, which occupies an area of 82 km^2^ [44], is mainly influenced by the East Asian monsoon system. During the warm season, the East Asian summer monsoon (EASM) circulation transports warm and moist air masses from the south or southeast to Rebun Island. A reversal of the major air pressure gradient forms the East Asian winter monsoon (EAWM) circulation, which is characterised by cold continental airflow from the north and the northwest. The Tsushima warm current (TWC) is another significant factor contributing to climate in the study region, flowing northwards along the eastern margin of the Sea of Japan. The summers are relatively mild and winters are cool. The annual mean temperature is 6.1°C. The monthly mean temperature drops below 0°C between December and March. During the coldest (January) and warmest (August) month, mean temperatures reach –6.4°C and 19.4°C, respectively [45].

Rebun Island is located within the cool mixed forest biome zone [46], characterised by cool temperate and boreal woody plants. The natural forest vegetation is dominated by boreal evergreen conifers and boreal and temperate deciduous broadleaf trees [43]. The natural forests on Rebun Island were largely cleared during the 20th century. Today, dense stands of *Sasa kurilensis* (dwarf bamboo) or *Reynoutria sachalinensis* (Sakhalin knotweed) dominate the vegetation cover and probably hinder the regeneration of arboreal taxa. However, several natural arboreal refugia still exist and are mainly found in valleys in the central and eastern parts of the island [43]. Here, a wealth of edible plants including *Sambucus sieboldiana* (elderberry), *Morus australis* (Chinese mulberry), *Actinidia arguta* (hardy kiwi), *Vitis coignetiae* (Crimson glory vine), *Juglans ailantifolia* (Japanese walnut), and *Phellodendron amurense* (Amur cork tree), can be found. *Empetrum nigrum* (black crowberry) grows on exposed locations around Mt. Rebun (Rebun Dake, Fig 1D).

## Material and methods

Okhotsk occupation at Hamanaka 2 is represented by a sedimentary succession, which is divided into eight stratigraphic units (IIIa–V, Table 1) defined on the basis of lithological characteristics and pottery typology. Based on pottery characteristics, the archaeological assemblage comprises the Okhotsk stages of the Susuya, Towada, Kokumon, Chinsenmon, and Motochi. To the best of our knowledge, the use of the water flotation technique at Hamanaka 2, as presented in this paper, is the first use of this archaeological method on Rebun Island. A total of 54 flotation samples from Okhotsk cultural layers were collected during the BHAP summer field excavations of 2013, 2014, and 2015 were analysed. Since the present study focuses on domesticated plants, we exclusively consider samples, which contain seed remains of cultivated cereals (*n* = 25).

**Table 1:** Hamanaka 2 cultural layers associated with the Okhotsk culture and available flotation samples.

Units	Sub-units	Okhotsk culture period (acc. to pottery finds)	Depositional context	Extracted flotation samples	
				Number	Volume in litres
III	IIIa	Motochi	Abundant fish bone remains	20	101.0
	IIIb	Chinsenmon, Motochi	Abundant fish bone remains	16	92.5
	IIIc	Chinsenmon	Abundant fish bone remains	9	98.0
	IIId	Kokumon, Chinsenmon	Abundant fish bone remains	6	24.0
	IIIe	Kokumon	Few fish bone remains	1	8.0
	IIIf	Kokumon	Abundant charcoal fragments	-	-
IV		Susuya, Towada	Few eroded fish bone remains	1	4.0
V		Towada	Abundant sea mammal remains, few fish bone remains	1	1.0

Extraction of the light fraction from sediments was performed using an overflow-style machine [47]. Seed and fruit parts were identified using morphological traits with the aid of a binocular microscope. Uncarbonised plant remains found in the context of prehistoric deposits may be intrusive, and therefore, we exclusively considered carbonised material. Highly fragmented seeds, in most cases comprising less than 50% of their original size, were not counted in this study (even if they were morphologically diagnostic), in an attempt to keep from counting seed fragments from the same seed more than once. The removal of small seed fragments from the totals prevents artificial inflation of the numbers and serves the same function as minimum number of individual estimates. Photographic documentation and morphometric measurements of the recovered barley grains were collected using a VH-Z20 R (x20–x200) zoom lens mounted on a Keyence VHX-1000 microscope. Biometric measurements, including maximum length (L), width (W), and thickness (T), were taken from intact seeds showing no or minor damage. The former two dimensions were measured with the seed dorsal side facing up. Weight was determined with a Mettler Toledo WXTS205DU precision scale. Age determination of Okhotsk culture units bearing carbonised barley seeds was performed directly on the seed material at the Poznan Radiocarbon Laboratory. To ensure reliable dating, each dated specimen had a weight of more than 2 mg. AMS ^14^C dates were calibrated to calendar ages using OxCal v4.2.3 software [48].

In order to trace the origin and to reconstruct potential pathways of dispersal for the barely that we recovered on Rebun Island, this study attempts the morphological correlation of the barley seed population from the Hamanaka 2 site with contemporaneous barley assemblages found in surrounding regions. Therefore, we compiled a dataset containing morphological measurements of ancient seeds available through published and unpublished macrobotanical records from archaeological sites in Hokkaido and adjacent regions including Tohoku (northern Honshu) and the RFE (Fig 1A–1C). The data were extracted from articles and published/unpublished excavation reports all written in either Japanese or Russian. These publications either provide length, width, and thickness (breadth) measurements for each (or a selection) of the extracted barley caryopses or give arithmetic means of these parameters for the analysed seed populations. To compare the morphology of different seed populations, various parameters based on the three-dimensional measures (i.e. L, W, and T) have been used (e.g. [49, 50]). Here, the ratio of length to width (L/W) is used to quantify the “plumpness” or compactness of the extracted barley seeds, in a manner consistent with previous studies on similar material from the wider study region (e.g. [15, 23]).

The vegetation reconstruction presented later in this paper is based on pollen analysis of the lake-bottom sediment from the 19.5-m-long core RK12 [43], which was retrieved in 2012 from Lake Kushu, located ca. 1.5 km east of the Hamanaka 2 site (Fig 1E). The chronology of the RK12 sedimentary succession is built on 57 AMS radiocarbon dates (see [43] for details regarding the core lithology and chronology). The high-resolution pollen analysis performed for this study is focused on the ca. 290-cm-long section, spanning an interval from the 1st to 15th century AD. The analysed sub-samples (each representing 1 cm core depth) were extracted from the core in 4 cm steps each equivalent to a time period of ca. 20 years. The sub-samples were processed in the chemical laboratory of the Paleontology Section at Freie Universität Berlin and microscopically analysed (see [51] for technical details and references for pollen identification).

## Results and interpretation

### Chronology

**Fig 2:**
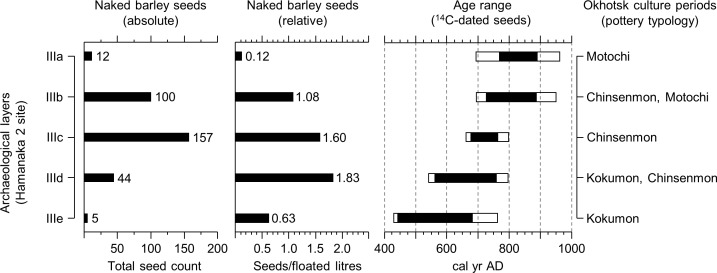
Summary of the barley assemblage in the Hamanaka 2, Rebun Island, northern Japan. Absolute and relative amounts of naked barley *Hordeum vulgare* var. *nudum*) seeds (incl. *Hordeum*/*Triticum* type) and identified pottery types per cultural layer. Given age ranges are based on radiocarbon dates of barley seeds (Table 2) with black and white bars indicating 68% and 95% confidence interval, respectively.

Of the eight Hamanaka 2 archaeological layers (IIIa–V) typologically associated with the Okhotsk culture (Table 1), five (IIIa–IIIe) contained carbonised barley remains. The AMS radiocarbon dates (Table 2) suggest the presence of barley at the Hamanaka 2 site during a time period of ca. 500 years. Given the 95% confidence interval of the oldest and youngest calibrated (INTCAL13; [52]) radiocarbon dates, this period is temporally bounded between ca. 430–960 cal yr AD. Application of the 68% confidence ranges of the INTCAL13 calibration curve narrows the time period to ca. 450 (ca. 440–890 cal yr AD) years. The results illustrate that the age ranges of the dated seeds are in line with the stratigraphic order of the identified Okhotsk cultural layers (Fig 2). Conservatively estimated age ranges (INTCAL13, 95% confidence interval) for layers IIIe, IIId, IIIc, IIIb, and IIIa are 428–765, 542–800, 661–800, 692–951, and 694–965 cal yr AD, respectively. This roughly corresponds to the Okhotsk culture stages associated with layers IIIa–IIIe dating between 6th and 10th century AD [27].

**Table 2:** Radiocarbon dates and calibrated ages on carbonised barley seeds from the Okhotsk culture layers of the Hamanaka 2 site, Rebun Island, Hokkaido.

Lab number	Sample name	Hamanaka 2 cultural layer	AMS radiocarbon date, uncal yr BP	Calendar age (OxCal v4.2.3, [48, 52], 95% range), cal yr AD	Calendar age (OxCal v4.2.3, [48, 52], 68% range), cal yr AD
Poz-84277	F2013-081-001	III a	1215 ± 30	694–889	769–874
Poz-84278	F2013-081-002	III a	1170 ± 30	771–965	777–892
Poz-81340	F2013-147-008	III b	1220 ± 30	692–887	726–870
Poz-81341	F2013-147-013	III b	1215 ± 30	694–889	769–874
Poz-81342	F2013-147-015	III b	1180 ± 30	730–951	777–886
Poz-84280	F2013-139-001	III c	1285 ± 30	665–771	678–766
Poz-84281	F2013-139-003	III c	1275 ± 30	661–800	685–766
Poz-84282	F2014-019-001	III d	1295 ± 30	662–770	672–765
Poz-84283	F2014-019-002	III d	1335 ± 30	646–766	652–758
Poz-84284	F2014-113-001	III d	1475 ± 30	542–644	560–620
Poz-84285	F2014-113-002	III d	1275 ± 30	661–800	685–766
Poz-84286	F2014-037-002	III e	1350 ± 30	636–765	650–680
Poz-84287	F2014-037-003	III e	1520 ± 30	428–609	438–596

### Macrobotanical analysis

This study focuses on samples from Okhotsk culture layers at the Hamanaka 2 site, which contain domesticated plant remains. In total, we have analysed 54 flotation samples from Okhotsk culture contexts, of which 25 contained remains of domesticated barely and/or barely/wheat-type seeds. Seeing that none of the well-preserved grains morphologically resembled wheat (*Triticum*), we assume that the poorly preserved grains all represent barley as well. From the samples, about 900 seeds and seed fragments were extracted and arranged into 22 categories. The assemblage also contains around 27 unidentifiable seed fragments and 67 unidentified seeds. The results of the macrobotanical analysis are outlined in the following section and in part listed in Table 3. Complete counting results are provided as a supplement (S1 Table).

**Table 3:** Sample-specific total counts of selected macrobotanical remains and floated litres from the Okhotsk culture layer units of Hamanaka 2.

Sample ID	Layer	Volume (litres)	*Hordeum vulgare* var. *nudum*	*Hordeum*/*Triticum* type	*Phellodendron amurense*	*Rhus/Toxicodendron* sp.	*Vitis* sp.	*Actinidia* sp.	*Malva* sp.	*Polygonum* sp.	*Chenopodium album*	*Cyperus* sp. (root tubers)	Unidentified seed	Unidentifiable seed fragments	Sum^a^
F2013–065	IIIa	14		1						4	16		1	1	22
F2013–071	IIIa	13		1	1					1	3		1		7
F2013–075	IIIa	13	1	1											2
F2013–081	IIIa	15	2			1		1			1		1		6
F2013–097	IIIa	15	3	1	6	8	1	6	1		1	2	3		32
F2013–137	IIIa	20	1		1			1		1					4
F2014–007	IIIa	11	1												1
F2013–129	IIIb	14	2	1								1			4
F2013–135	IIIb	10	11	8	1	20		4				1	3	3	48
F2013–145	IIIb	13	13	5		47	3	9		7	8		11		103
F2013–147	IIIb	11	24	3	15	66	67	82	2	6	4		16	12	285
F2013–153	IIIb	10	16	3	4	12	1	5		2	3	1	3		50
F2014–005	IIIb	12.5	2	1											3
F2014–009	IIIb	10	4	6		38		2					2		52
F2014–059	IIIb	12	1												1
F2013–095	IIIc	15	5	3	1	12	2						1	2	24
F2013–139	IIIc	15	16	8		1				1			3	1	29
F2013–141	IIIc	15	15	9		3	1	5		1	5		1	1	40
F2013–155	IIIc	16	12	5			1	1		1	4		3		27
F2013–161	IIIc	10	14	6	1	6	1	2			5		8		43
F2013–163	IIIc	15	1	1		3						2			7
F2014–011	IIIc	12	57	5		8			5			2	9	4	86
F2014–019	IIId	12	19	9		1				1	1			3	31
F2014–113	IIId	12	10	6		39				1					56
F2014–037	IIIe	8	3	2									1		6
**sum**		**324**	**233**	**85**	**30**	**265**	**77**	**118**	**8**	**26**	**51**	**9**	**67**	**27**	**969**

Unless otherwise stated, the shown plant taxa represent seed remains.

^a^ not including fragments

#### Wild plant seeds and tubers

A total of 604 complete or fragmented seeds and root tubers of wild plants were identified. The assemblage comprises plant remains that we interpret as a foraging component of the Okhotsk culture subsistence economy. This includes carbonised remains of *Phellodendron amurense*, *Vitis*, *Actinidia*, *Polygonum*, *Chenopodium album*, Amaranthaceae, *Sambucus*, and *Rubus*. Most of these plants are still abundant in undisturbed natural vegetation refugia on Rebun Island. Several charred root tubers were found in the samples, which we associate with plants contained in the genus *Cyperus*. This suggests that root components of nutsedges were another food source of the local Okhotsk people. The analysed samples also contained seeds of ruderal plants, which may indicate human-induced disturbance at the Hamanaka 2 site, for example, *Malva*, *Rumex*, and Poaceae.

#### Barley seeds

**Fig 3:**
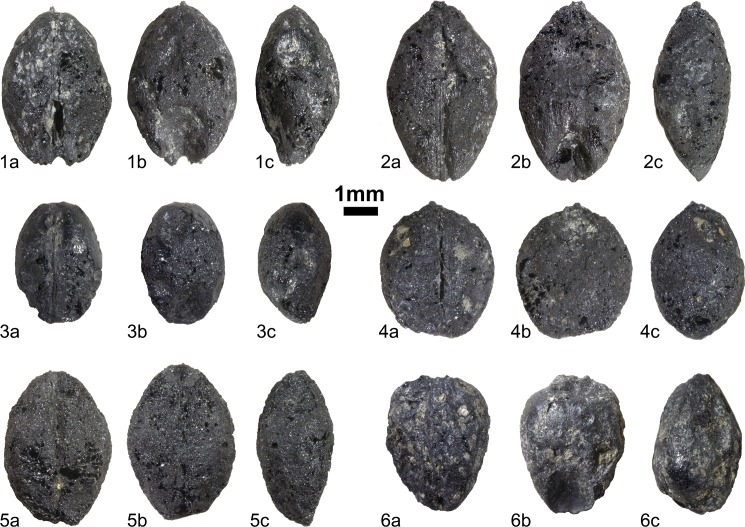
A selection of compact naked barley grains from Okhotsk culture layers of the Hamanaka 2 site, Rebun Island, northern Japan. Depicted grains are representative of the barley-bearing cultural layers (IIIa–e) covering the time period ca. 430–960 cal yr AD. Each of the six specimens is shown in ventral (a), dorsal (b), and lateral (c) views.

While there were abundant seeds of wild plants, many of which have economic significance, the barley remains are the only domesticated plant contained in the Hamanaka 2 assemblages. Morphological traits such as maximum width at the grain’s centre, a roundish shape in cross-section, rounded ends (as seen from the dorsal or ventral side), and a relatively wide ventral furrow (Fig 3) detected on fully intact or weakly fragmented specimens suggests that they are six-rowed naked barely (*Hordeum vulgare* var. *nudum*). In total, we confidently identified 233 carbonised grains as naked barley. Due to poor preservation, another 85 cereal grains were simply referred to as *Hordeum/Triticum*-type, though they are likely barley, since no wheat grains were identified. The total abundance of naked barely grains (incl. *Hordeum/Triticum*-type) increase from layer IIIe (n = 5) to IIId (n = 44), are most abundant in IIIc (n = 157), and then decrease in IIIb (n = 100) and IIIa (n = 12) (Fig 2). To calibrate for the volume of soil processed in each layer, we plotted the absolute numbers in relation to floated litres of sediment (density ratios). This shows that the highest seed concentration is found in layer IIId with 1.83 seeds/litre, which decreases towards the topmost layer (IIIa) represented by a value of 0.12 (Fig 2).

We measured 87 well-preserved barley caryopses for length (L), width (W), and thickness (T) dimensions (S3 Table), with mean and median values of 4.13, 2.98, and 2.32 mm and 4.10, 3.00, and 2.30 mm, respectively. Given these measurements, biometric parameters like L/W (mean = 1.39, median = 1.39) or L/T (mean = 1.79, median = 1.77) ratios, which may be used to correlate the morphology of different populations, were inferred. As potentially useful in quantitative approaches [53], we also determined the weight of each well-preserved grain. However, all of these values, especially weight, are highly influenced by taphonomic processes and the carbonisation process, leading to highly distorted or less-dense grains.

### Analysis of barely morphological data

**Fig 4:**
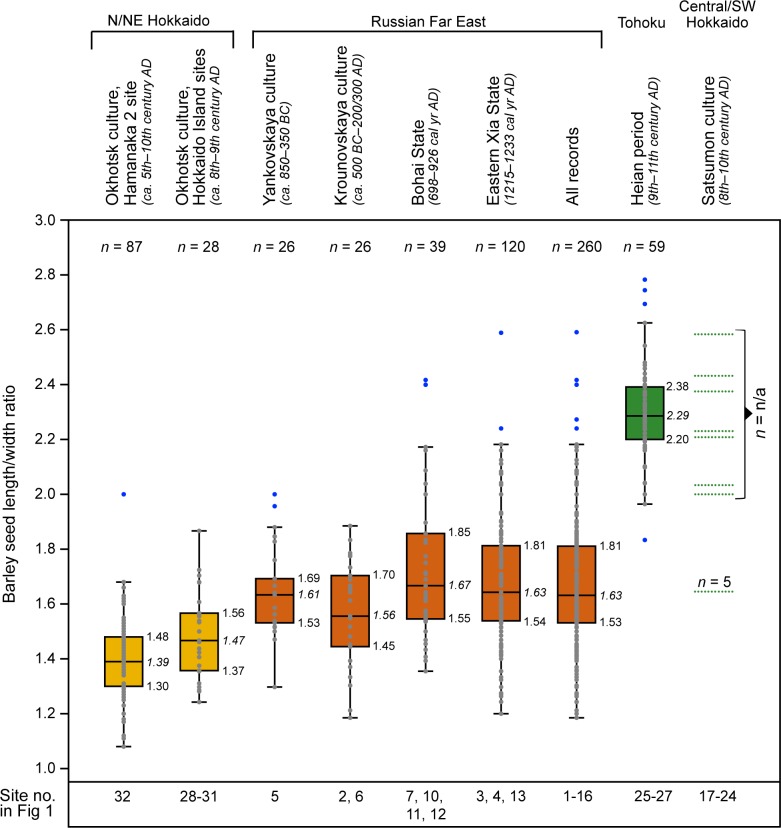
Box-plots showing the L/W distribution of barley seeds from the Hamanaka 2 Okhotsk culture layers (this study) and of selected records from other regions. The boxes delineate the 25–75% quartiles (regular font) with the median (italic font) shown as an inset horizontal line. The whiskers (inner fence) are defined as the array from top/bottom of the box to the largest/smallest data point less than 1.5 times the box height from the box. Data points inside and outside (outliers) the whiskers are marked by grey and blue dots, respectively. Dotted horizontal lines indicate arithmetic means of Satsumon barley populations. Sample size (if know) and site numbers used in Fig 1 are provided. Ages are given in calendar years.

In order to track the provenance of the barley used by the Okhotsk culture people we have compiled barley morphological data from archaeological excavations in adjacent regions, comprising 15 sites located in northern Japan, including Hokkaido and northern Tohoku, and 16 sites in the RFE (Fig 1A–1C; S3 Table). The distribution of the L/W ratios of selected records are summarised as box-plots in Fig 4. A complete summary of the collected data including references is available as supplementary material (S3 Table).

In addition to the barley morphological measurements from Hamanaka 2 (S2 Table), the data set contains data from four other Okhotsk sites (Fig 1C, no. 28–31) dated between the 5th and 9th century AD (S3 Table). For two assemblages (*n* = 26) seed-specific length, width, and thickness measurements are available. Calculation of the median for the L/W ratio yield a value of 1.47 and 1.49, respectively. For the other two sites, which comprise 30 and 29 barley grains, respectively, the arithmetic means for the L, W, T and L/W (1.56 and 1.30, respectively) are based on published information. The L/W ratio of barley from all four sites equals 1.49.

Within the RFE, 15 sites are situated in southern Primor’e (Fig 1B, no. 2–16) and one in the western Amur River valley (Fig 1A, no. 1). The Primor’e sites are associated with early Iron Age Yankovskaia (ca. 850–350 cal yr BC) and Krounovskaia (ca. 500 cal yr BC–200/300 cal yr AD) cultures, the Iron Age Ol'ginskaia culture (ca. 300 cal yr BC–300/400 cal yr AD), the early medieval Mohe culture (ca. 5th–11th century AD), the Bohai State (698–926 cal yr AD), the period following the defeat of the Bohai State (10th century AD), and the Eastern Xia State (1215–1233 AD). The barley finds from the western Amur River valley site represent Troitskii variant of the Mohe culture (end of 8th–9th century AD). From these sites there are in total 25 barley records of which 17 contain measurement data for individual grains. The number of grains per record ranges from 1 to 40. For the remaining eight barley assemblages only arithmetic mean values are provided, without the total number of grains reported. Mean values for L/W of all 25 records range between 1.27 and 2.13. The average of all arithmetic means is 1.67. To minimise the weight of outliers, median values were calculated for grain sets (*n* = 16) containing measurements of at least four specimens. The results range between 1.45 and 1.88. The L/W median of all specimens (*n* = 260) for which individual measurements are available is 1.63.

A total of eleven samples of archaeobotanical barley from south of the Okhotsk culture domain were also considered in this study. They originated in the Hokkaido Region from Early to Middle Satsumon culture (Fig 1C, no. 17–24) and northern Tohoku Heian period (Fig 1C, no. 25–27) sites and date to 8–10th and 9–11th centuries AD, respectively. Scholars have suggested that during the Okhotsk Tobinitai stage (11th–12th century AD; [26]), which emerged in the north-eastern part of Hokkaido, there were enhanced interactions with Late (ca. 1000–1200 cal yr AD) Satsumon groups [54]. Therefore, the review of Satsumon barley is limited to the early and middle stages. The information available for the Early and Middle Satsumon sites (*n* = 8) is restricted to mean values for L/W ratios, which vary between 1.65 and 2.58 with an average of 2.16. Absolute numbers of measured seeds are not provided in the publications, except for one site (Fig 1C, no. 24). Information from northern Tohoku is based on measurements of three assemblages containing 2, 7, and 50 barley specimens. Their length to width ratios equate 2.3, and their median is 2.29.

For Early and Middle Satsumon barley assemblages information on grain shape (i.e. L/W ratio) is only available in the form of arithmetic means, a statistic which is sensitive to outliers. However, these values are, with one exception, all within the inner whiskers of box-plots of the L/W values for barley from northern Tohoku sites (Fig 4), thus regarded as representing comparable barley varieties. One notable exception is the relatively low mean value of 1.65. Given its small population of five grains and strong offset to the other means, this sample may be regarded as an outlier. The results delineate that the charred barley seeds recovered from Okhotsk culture sites are the most compact. Although still plumper, they are more comparable in shape to grains from the RFE than to those found in Satsumon sites on Hokkaido and Heian period sites in northern Tohoku. While the barley from south of the Okhotsk domain appears to be longer and narrower, the Okhotsk culture and RFE varieties are more compact. The differences in L/W ratio indicate that the long grains comprise hulled and the compact ones naked barley. The '*nud*' allele for naked barley is monophyletic [55], thus genetically distinct from hulled barley. This implies a sharp difference between the barley that was grown in these different regions.

### Local vegetation reconstruction

**Fig 5:**
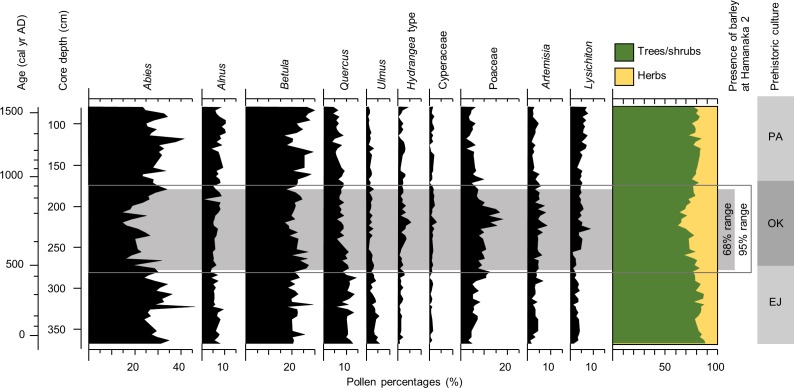
Simplified pollen diagram representing the section dating between 50 cal yr BC and 1540 cal yr AD (67 pollen spectra) of the sediment core RK12 from Lake Kushu, Rebun Island. Chronology of prehistoric cultures in northern Hokkaido comprises Epi-Jomon (EJ; ca. 100 cal yr BC–500 cal yr AD including the Susuya tradition ca. 100–500 cal yr AD), Okhotsk culture (OK; ca. 500–950 cal yr AD), and Proto-Ainu/Formative Ainu (PA; ca. 950–1600 cal yr AD) (according to [26] and [27]).

The pollen analysis results of a ca. 290-cm-long section (369.5–76.5 cm) of the RK12 sediment core from Lake Kushu (Fig 1E) allows a high-resolution insight into changes in vegetation cover on Rebun Island during the 1st–16th century AD. Lake Kushu has a small surface area of 0.5 km^2^ and hosts an excellent sedimentary archive of the local vegetation, climate, and human impacts on the island’s ecosystem [44]. Except for neighbouring Rishiri Island, the closest pollen source area outside Rebun Island is the main island of Hokkaido situated 45 km to the east. Thus, pollen influx into the lake may be regarded as a local vegetation signal confined to Rebun Island with minor disturbance of far-distant transported pollen [56]. Based on the composition of the pollen assemblages, the analysed core section may be divided into three main phases, which are broadly synchronous with the main cultural phases of the study region (Fig 5).

The interval prior to ca. 400 cal yr AD in the pollen record is characterised by a high average proportion of arboreal (tree and shrub) taxa, reaching 82% of the total terrestrial pollen sum. *Abies* (fir) and *Betula* (birch) contribute the highest percentages to the pollen assemblage, i.e. around 30% and 22%. Percentage values of herbaceaous taxa like Poaceae (grasses), Cyperaceae (sedges), *Artemisia* (wormwood), and *Lysichiton* (Asian skunk-cabbage) are relatively low during this phase with mean values of 5%, 1%, 3.5%, and 3%, respectively.

The interval between ca. 400–900 cal yr AD reveals substantial decrease in the arboreal pollen taxa percentages compared to the previous interval (Fig 5). *Abies* pollen percentages range on the low level of around 20%. Especially low abundances (19%) are evident at between 225.5 and 189.5 cm (640–800 cal yr AD). Slightly reduced values (20%) for this period are also obtained for *Betula*. On the other hand, *Hydrangea* type pollen are elevated, which we interpret as an increase in shrubby Hortensia plants around the study site. Unlike *Abies* and *Betula*, percentages of *Quercus* (oak) and *Ulmus* (elm) pollen do not show a decline, while the contributions of herbaceous taxa including Poaceae and *Artemisia* increase. In particular, Poaceae percentages increase to 10–18%.

During the ca. 900–1540 cal yr AD interval, the pollen assemblage again shows relatively high proportion of arboreal taxa (ca. 80%, i.e. ca. 10% higher than during the peak of the Okhotsk occupation), suggesting that forests (and particularly fir stands) on the island fully recovered after 800 cal yr AD. *Abies* pollen contribution reached ca. 42% around 1375 cal yr AD (117.5 cm), while Poaceae percentages return to ca. 5%.

We interpret the combination of the outlined changes in pollen abundances as a result of human activities leading to a more open vegetation cover. This is mainly reflected by the decline of *Abies* and *Betula*, which promoted a contemporaneous spread of herbaceous (Poaceae and *Artemisia*) and shrubby taxa (*Hydrangea*) on open landscapes. A significant climate shift towards cooler and dryer conditions–another frequently involved factor of the described changes in vegetation–can be virtually excluded, since the thermal and moisture conditions of the island [45] greatly exceed requirements of boreal tree taxa, such as *Abies* and *Betula*. The increased contribution of *Lysichiton* pollen from 2.8% prior to 400 cal yr AD to 4.5% after could be a further indication of human activities in the area. Asian skunk-cabbage is a plant whose above-ground parts are edible (when cooked) and which is typically found in swampy environments. It was naturally growing along the shore of Lake Kushu. We hypothesise that the local community managed the shore vegetation in favour of *Lysichiton* growth to obtain supplementary food sources. Our pollen-derived reconstruction of the local vegetation suggests stronger human impact on the island vegetation between ca. 400 and 900 cal yr AD, corroborating the archaeological data for the regional Okhotsk culture occupation phase.

The results of the pollen analysis also indicate that the reconstructed phase of intensified human impact was superimposed onto a long-term climatic cooling trend, suggested by decreasing percentages of *Quercus* and *Ulmus*. Spanning the entire record, both of these thermophyllous taxa show a continuous gradual decline from roughly 10% and 5% at the bottom of the record to about 5% and 1.5% at the top, respectively. This trend likely indicates a progressive cooling in the study region in line with the late Holocene trends of Northern Hemisphere summer insolation [57] and published palaeoclimatic records from the region (e.g. [58, 59]).

## Discussion

There is evidence that human migrations from the north have played an important role in the prehistory of Hokkaido and other parts of the Japanese archipelago. This includes the intrusion of Siberian Palaeolithic hunter–gatherer groups around the Late Glacial Maximum (ca. 20,000 cal yr BP; [60]) and immigration ca. 15,000 cal yr BP, with the latter introducing microblade technologies on Hokkaido and Honshu [61, 62]. While they have not been taken into account for a while (e.g. [63]), recent anthropological studies (e.g. [64]) stress the role of migration from northern regions via Hokkaido also in view of the origins of the Neolithic Jomon culture. The most recent southward movement of prehistoric populations into the northern and north-eastern coastal areas of Hokkaido was that of the Okhotsk culture around the middle of the 1st millennium AD [26]. Though, the Okhotsk groups inhabited a large area along the southern and eastern margins of the Sea of Okhotsk, most of our current knowledge has been derived from archaeological materials recovered in the Hokkaido Region. The archaeobotanical record from the Hamanaka 2 site presented in this study allows for greater insight into the use of plants by the Okhotsk people on Rebun Island (Fig 1D). Calibrated ages (95% confidence interval) of directly dated barley remains from five archaeological layers (IIIa–e) suggest that the crop was used at the site between 430–960 cal yr AD (Table 2) or at a 68% confidence interval between ca. 440 and 890 cal yr AD. This time period roughly corresponds to the late Susuya through mid-Motochi stages spanning between the 5th and 10th century AD [27], thus covering the Okhotsk culture settlement phase in northern Hokkaido as indicated by previous archaeological studies. Given the age of the oldest barley seed F2014-037-003 (440–600 cal yr AD, 68% confidence interval; Table 2), the Hamanaka 2 layer IIIe, together with the single dated grain (428–573 cal yr AD, 95% confidence interval; [65]; S3 Table) from the Oumu site (no. 29 in Fig 1C) represents, the earliest well-documented record of domesticated barley in the Hokkaido Region. The only carbonised barley grain recovered in Hokkaido was collected from the Epi-Jomon level of the K135–4 Chome site within the city of Sapporo [66]. This single barley seed has not been directly dated and its proposed age of ca. 200–400 cal yr AD should be viewed with caution.

The presence of barely at the Hamanaka 2 site appears contemporaneous with a phase of enhanced human-induced vegetation disturbance on Rebun Island as indicated by the pollen record from Lake Kushu (Fig 5). During this time (with a maximum ca. 550–800 cal yr AD), the pollen record shows a decrease in the abundance of arboreal pollen, suggesting deforestation and greater openness of the landscape compared to the preceding and subsequent periods, which are more or less coeval with the Epi-Jomon and Proto-Okhotsk (Susuya) cultures (ca. 100 cal yr BC–500 cal yr AD) and Proto-Ainu culture (ca. 950–1600 cal yr AD), respectively ([26, 27]; Fig 5). The results of the local vegetation reconstruction clearly indicate enhanced human activities on Rebun Island during the main phase of the Okhotsk presence there. On the other hand, reduced impact is evidenced during the Epi-Jomon phase and the time of cultural shifts towards the Classic Ainu period, which may be explained by reduced population size and/or a different pattern of resource exploitation. Regarding the Epi-Jomon, this would conform to identified traits like short-term occupations, high mobility, and low complexity [66, 67]. Ohyi [28] suggests that by the time of the disappearance of the Okhotsk culture at the end of the Motochi stage, the Satsumon people spread into northern Hokkaido, including Rebun Island and neighbouring Rishiri Island. It appears, at least for Rebun Island, that these Satsumon groups weakly impacted the island’s vegetation, which was leading to the recovery of local fir forests. Fewer human activities at Hamanaka 2 are also suggested by the absence or quantities of archaeological materials (unpublished data) associated with the Satsumon culture in the subdivisions of layer II.

Rebun Island is well-known for its Okhotsk culture sites, representative for the northern Hokkaido domain. Here, the presence of the Okhotsk groups continued into the Motochi stage (9th–10th century AD) at a time when the Okhotsk sites in northern Hokkaido became abandoned [28]. However, on Rebun Island the presently discussed Hamanaka 2 assemblage contains the only barley thus far recovered (T. Amano, personal communication). This might be due to a lack of systematic sampling and water flotation at other sites on the island. We identified the recorded barley as naked barley, which is far more commonly found than hulled barley in East Asia [68]. Barely was consumed at the site over a ca. 500-year period throughout the main stages of the Okhotsk culture (Fig 5). Barley was significant and had a long-term role in diet during the peak of the Okhotsk culture in the region. The use of barley is also evident at other sites in north-eastern Hokkaido (Fig 1C), being assigned to the late phase (8th–9th century AD) of the Okhotsk culture [69]. In addition, remains of foxtail and broomcorn millet are reported from several excavations [69]. Japanese palaeobotanists have argued that these crops were used for ritual purposes (e.g. [22]); however, this is hard to defend seeing that they appear in so many domestic contexts across such a large time period. The grains likely supplemented a mixed economic system that relied heavily on wild coastal resources. Although, an alternative hypothesis is that these crops were used to produce alcohol [70]. Another important question to ask is whether barley was obtained by trade or cultivated locally by the Okhotsk people or a combination of both. At Hamanaka 2, no tools related to barley cultivation or processing have been found and no tests of organic residue on pottery have been conducted yet to support either use. Yamaura [71] argues that the axe- and hoe-shaped bone tools unearthed in eastern and northern Hokkaido Okhotsk sites (including Kafukai A, southern Rebun Island) represent evidence for crop cultivation. However, this is not conclusive evidence, since such tools might have had other purposes like collecting wild plants and digging dwellings and/or storage pits.

One reason why some scholars have been hesitant to accept that barley and other cereals were dietary supplements may be that the Okhotsk are generally regarded as a specialised hunter–gatherer culture with a subsistence strongly focusing on maritime food resources. This traditional view of a coastal foraging society has been bolstered by recent human bone isotope studies (e.g. [35, 38, 39]), which revealed a high proportion of absorbed protein derived from marine resources of up to 94% and 90% in northern and eastern Hokkaido, respectively [39]. However, our findings together with results of previous studies illustrate that the Okhotsk relied on a wide range of natural and domesticated foods. Besides the suggested strong focus on marine collecting, fishing, and mammal hunting, the Okhotsk people appear to have employed a broad spectrum of wild terrestrial plant fruits and root tubers (this study, Table 3; [26] and references therein), hunted a variety of terrestrial mammals [37], and also maintained domesticated dogs [36] and pigs [26] as part of their food economy. Indications for plant maintenance also comes from the increase in *Lysichiton* type pollen in the Lake Kushu pollen record (Fig 5) and carbonised *Cyperus* sp. root tubers in the Hamanaka 2 flotation samples (Table 3). Both taxa represent plants growing in swampy environments around Lake Kushu, which include edible parts and provide nutritious food. It is conceivable that the local Okhotsk people exploited these plants and even maintained their growth and productivity using suitable tools for tilling as found in contemporaneous cultural strata on Rebun Island [71].

Given the combination of foraging, animal husbandry and the use of barley and other cereals over a wide spatio-temporal array, crop cultivation as a supplementary portion of Okhotsk subsistence seems more plausible. This case study further augments existing examples of (“complex”) hunter–gatherers, occupying the “middle ground” which separates hunting–fishing–foraging societies exclusively depending on wild food resources and agriculturalists with a major focus on managing and producing domesticated plants and animals (e.g. [72, 73]). It has been noted that this middle ground territory is highly complex. As Smith [9] puts it, “this territory between hunting–gathering and agriculture is turning out to be surprisingly large and quite diverse; it has also proven to be quite difficult to consistently describe in even the simplest conceptual or developmental terms”. Smith [74] built his concept of ‘low-level food production’ on earlier observations by Braidwood and Howe [75] as well as Flannery [76], all of whom use the term “incipient cultivation” to describe intermediary strategies between foraging and farming. Many other ethnographers and archaeologists have subsequently noted that there is a wide range of diversity in human economic systems; notably, Boserup [77] points out that the range of land-use strategies reflect an equally broad range of human adaptive economies. There have been different approaches to define the middle ground landscape. Following the conceptual framework of Smith [9], who identified low-level food production (<50% annual caloric budget from domesticates) relating to tended wild plants and/or cultivated/managed plants. The Okhotsk culture may be confidently placed somewhere between the traditionally accepted boundaries between foraging and agriculture. The same view is taken by Crawford [8] who explicitly assumes that the Okhotsk people themselves cultivated barley and millet. Given the evidence for dog and pig husbandry and the cultivation or exchange of barley, the Okhotsk epitomises the complexity and diversity of the middle ground economy. Smith [9] illustrates that for many prehistoric societies this in-between space did not mark a brief transitional phase towards agriculture, but a state of stable long-term (often over several millennia) or even permanent economies combining “low-level production” of domesticates and a major focus on wild resources. Such more or less stable “mixed” subsistence economies are also evidenced in a number of modern and historic hunter–gatherer groups and are mostly characterised by either the adoption of cultivation or the exchange of domesticates (e.g. [78] and references therein). In light of the present evidence, it appears that also the Okhotsk people employed such socio-political and economic strategies.

Thus far, the ongoing debate about the middle ground landscape has either been centred on early “mixed” subsistence economies (e.g. in New Guinea [7] or eastern North America [4]) or on recent ethnographically observed cultures (e.g. the Mikea people of Madagascar [79] or the Agta of the Philippines [80]). In this regard, the adoption of domesticates by the Okhotsk people, which occurred in relatively recent prehistory, adds particular value as it bridges the gap between the two foci. Another specific feature of the Okhotsk subsistence strategy is the process of adopting already fully domesticated plants that were, by this time, widely used as staple crops in agrarian societies across Eurasia. In the Okhotsk culture, however, the incorporation of barley and millets does not appear to have had significant socio-economic effects. While the subsistence economy continued to be based on foraging, the society remained egalitarian and “group-oriented” [26]. Similar observations are reported from other regions like Japan as well as Island Southeast Asia and Melanesia. The spread of domesticated rice through the latter two regions appears to have started at the beginning of the 1st millennium BC [81]. However, in most regions, rice remained a minor supplementary crop in subsistence systems mainly based on vegeculture (e.g. taro, banana, and sago production) and foraging [82] until the middle of the 1st millennium AD [83]. In Japan, the oldest botanical remains of domesticated broomcorn and foxtail millet, barley, and rice date between the Middle and Late Jomon periods [17], thus may also indicate an early (pre-Yayoi) introduction of domesticated cereal crops from outside the archipelago as minor subsistence supplements not signifying a fundamental change in dietary pattern. All these examples immediately prompt the urgent question of what social or environmental conditions govern the decision of vegeculturalists and/or foragers to adopt domesticated seed crops? While progress has been made in addressing this central issue in anthropological and archaeological research (e.g. [84]), a more universal conceptual framework, if at all possible, has thus far not been suggested.

Another feature of the analysed flotation samples that raises questions is the abundance of *Rhus/Toxicodendron* seeds. There is ongoing debate about the taxonomic standing within these genera; while some scholars have treated *Toxicodendron* as a separate genus, others have treated it as a subgenus of *Rhus* (see [85] and references therein). In some regions plants of the *Rhus* complex have a long history as a medicine or spice [86]. Significant numbers of *Rhus/Toxicodendron* seeds were recovered from Jomon sites in southern Hokkaido [8]. In this record at least two *Rhus/Toxicodendron* species were found, one of which was identified as *Toxicodendron vernicifluum* (lacquer tree), which is related to lacquer production [8] and medical effects [86]. Lacquer items were major trade goods and symbols of authority throughout the past four millennia in East Asia. An example showing the value of such items from neighbouring regions are lacquer artefacts recovered from elite tombs at major urban centres such as Erlitou [87] in China dating back to the period that most Chinese scholars call the Shang (late second millennium BC). Today, three species of *Rhus/Toxicodendron* grow in the Hokkaido Region including *Toxicodendron trichocarpum* (syn. *Rhus trichocarpa*), *Toxicodendron orientale* (syn. *Rhus ambigua*), and *Rhus javanica* [88]. The *Toxicodendron* species (i.e. *T*. *vernicifluum*, *T*. *trichocarpum*, and *T*. *orientale*) contain toxic substances that can cause severe allergic dermatitis by direct contact with its plant parts or exposure to smoke or fumes from burning plant parts. Based on the pericarp cross section structure, Yoshikawa and Ito [89] have proposed a method to distinguish carbonised seeds of *T*. *vernicifluum*, *T*. *trichocarpum*, *T*. *orientale*, and *R*. *javanica*. However, application of this method failed to differentiate the *Rhus/Toxicodendron* seeds extracted from the Hamanaka 2 site flotation samples. Further research is needed to facilitate robust identification of carbonised *Rhus/Toxicodendron* seeds to species level and to understand the meaning (e.g. medical effects such as anti-inflammatory, antimicrobial, and antiviral [86]) of *Rhus/Toxicodendron* species for prehistoric people.

The adoption and dispersal of domesticated plants is a central topic of archaeological research and an important issue in understanding agricultural developments in different parts of the globe. So far, the assemblages from Okhotsk and Satsumon sites in Hokkaido represent the north-eastern edge of prehistoric barley dispersal across Asia. The upper end (600 cal yr AD) of the calibrated age range (68% confidence interval) of the oldest barley seed contained in the dated Hamanaka 2 sample set is coeval with the onset of the Satsumon culture (beginning 7th century AD), which is believed to have arisen from the Tohoku Region (Fig 1A) Yayoi culture populations driven to Hokkaido by expansion of the first Japanese state [8]. Therefore, the most straightforward inference would be that the barley used by the Okhotsk was derived from Satsumon groups spreading into the central and south-western part of Hokkaido. In fact, previous palaeobotanical work points to a different origin that is further emphasised by the Hamanaka 2 barley seed inventory. In previous studies, Japanese scholars claimed to have identified a short and a long barley type at Okhotsk and Satsumon culture sites in the Hokkaido Region, which they assigned to the crop’s naked and hulled form, respectively (see [69] and references therein). Based on this differentiation and seed morphology, Yamada and colleagues (e.g. [22, 23, 69, 90]) have hypothesised that Okhotsk barley originated from neighbouring regions on the Asian mainland. They found that the highly compact (naked barley) specimens extracted from four Okhotsk culture sites (no. 17–20 in Fig 1C, S3 Table) are distinct from the slimmer (hulled) barley (dated to 8–10th century AD) used by Early and Middle Satsumon groups, but similar to grains identified as naked barley found in the early Iron Age to medieval (ca. mid-1st millennium BC–early 13th century AD) sites in southern Primor’e (RFE). Their morphological comparison of barley grains is based on a length/width ratio dataset. Here, we review their approach by supplementing the available datasets, with eleven (partly unpublished) assemblages from the RFE, three records from northern Tohoku, and the measurements of the Hamanaka 2 site barley (S3 Table). Although naked barley is in general more compact than its hulled counterpart, quantitative morphological comparison allows for objective qualification of the recorded archaeological barley and may provide further confirmation for the proposed Okhotsk barley origin. The Okhotsk barley, which appears to be the most compact of all gathered records (Fig 4, S3 Table), in terms of shape is more similar to its counterparts found in both earlier and later (ca. 850 cal yr BC–1000 cal yr AD) sites located along coastal regions across the Sea of Japan (i.e. southern Primor’e, Fig 1C) and in the Amur River basin (Fig 1A), as opposed to grains recovered from contemporary Satsumon culture sites situated in central and southern Hokkaido. Both the Satsumon and Heian grains, which are morphologically similar to each other, appear generally longer and narrower than those used by the Okhotsk culture. This corroborates Crawford’s hypothesis that the Satsumon culture emerged from the Tohoku Yayoi culture [8], which, when being forced to migrate to Hokkaido, brought their barley with them. Alternatively, this similarity may at least suggest cultural interactions between Satsumon populations and communities in Tohoku. The results also corroborate the hypothesis that the Satsumon type barley represents hulled barely and that the naked Okhotsk barley originated in the continental RFE Region. The minor discrepancies in L/W ratios between the naked barley from Okhotsk sites and sites in the RFE (Fig 4) may reflect morphological differences commonly existing among landrace varieties of crops [91] or may be the result of different environmental conditions or irrigation [92]. This means that the barley used by the Okhotsk culture was either derived by exchange with continental populations (e.g. Mohe culture, Bohai State) from across the Sea of Japan or was brought along and cultivated by the Okhotsk culture from their region of origin (i.e. the lower Amur River basin). Unfortunately, no palaeobotanical studies have been conducted in the lower Amur River basin or on Sakhalin Island, which would have allowed us to trace back potential pathways of a southward barley introduction to Hokkaido. Further evidence for the existence of two barley phenotypes in Hokkaido comes from sites, which post-date the main phase of Okhotsk culture. Both types—the compact (naked) barley found at Okhotsk culture sites and the slim (hulled) barley found at Early to Middle Satsumon sites—are represented in archaeobotanical records from this time indicating that Okhotsk type naked barley cultivation/use continued during times of acculturation (i.e. Tobinitai culture) and the Late Satsumon stage [23]. In sum, these findings suggest that naked and hulled barley spread eastward through Asia and were introduced into the Japanese archipelago via different routes. While the area where hulled barley is recovered parallels the distribution of the Yayoi culture (south-western and central Japan) and the Satsumon culture (south-western and central Hokkaido), naked barley possibly propagated from Primor’e and adjacent regions during the Okhotsk culture spread, into Sakhalin Island and northern/north-eastern Hokkaido (Fig 1A). After the two barley phenotypes were independently introduced to Hokkaido, the boundary between both barley domains (Fig 1A) possibly existed for about 400 years across the island region until the beginning of the assimilation/replacement of Okhotsk populations by the Satsumon culture groups (ca. 1000 cal yr AD; [26, 27]).

Further evidence for a close relationship between the Okhotsk culture and Iron Age/medieval populations from the East Asian continent comes from analogies in subsistence economy as described by Sergusheva and Vostretsov [15]. Like the Okhotsk, the Yankovskaia culture (ca. 850–350 cal yr BC; [15]), which inhabited the coastal regions of today’s northern Korea and southern Primor’e, based its subsistence mainly on a wide range of marine resources. Remains of millets (broomcorn and foxtail) and naked barley found at the majority of sites suggest that these domesticates also played a role in diet there. Primor’e Region, in particular, saw a subsequent two-step advance in agricultural practices by the introduction of additional crops including wheat (*Triticum aestivum*/*compactum*), hemp (*Cannabis sativa*), and legumes during the Krounovskaia/Tuanjie culture (ca. 500 BC–200/300 cal yr AD; [15]) and cultigens including hulled barley (*Hordeum vulgare*), soy beans (*Glycine max*), and buckwheat (*Fagopyrum esculentum*) along with the establishment of early states (e.g. Bohai State) after the middle of the 8th century AD. Despite this progress in agriculture, hunting, fishing, and gathering has continuously been an additional part of the diet. In addition, pig and dog breeding is evidenced during the Yankovskaia culture and the Krounovskaia/Tuanjie culture [71, 93, 94]. These subsistence traits place the above mentioned cultures of coastal RFE and northern Korea also in Smith’s middle ground [9], thus somewhere between hunting–fishing–foraging and agriculture. Systematic water flotation has been practiced for over 30 years in this region, providing archaeobotanical remains from numerous Neolithic–Middle Age sites across Primor’e. These data show that during the Yankovskaia culture, millet and barley cultivation was not the main part of the food economy, and was probably not practiced at every archaeological site [15]. Although crop cultivation seems to have been intensified by the Krounovskaia/Tuanjie culture, there is evidence that these groups, likely due to climatic cooling at the end of the 3rd century AD, partly gave up agricultural practices and re-intensified the exploitation of wild resources [95]. This, on the one hand, emphasises that the transformation towards agriculture is not necessarily a unidirectional progression, as it was once regarded, but is a reversible process. On the other hand, it suggests that crops probably had a long-term utility as complementary foods. This might apply to Okhotsk groups, which retained their once-adopted (low-level) agricultural food production as they migrated and adapted to the maritime landscapes of the Sea of Okhotsk.

## Conclusions

The archaeobotanical assemblage from Okhotsk cultural layers at the Hamanaka 2 site (northern Rebun Island, Japan) contained charred grains of compact naked barley. Direct radiocarbon dating indicates long-term use of barley at the site over a period of about 500 years. Together with the finds from the Oumu site, the data that we present marks the oldest well-documented evidence for the use of barley in the Hokkaido Region. Due to the broad error ranges of the calibrated radiocarbon dates of the oldest seed remains (428/440–573/600 cal yr AD, 68% confidence interval), more precise ages cannot be defined at this time. However, it is conceivable that the people of the Okhotsk culture were using this crop since they first arrived in the Hokkaido Region (ca. 500 cal yr AD). Accordingly, barley introduction by the Okhotsk culture would pre-date its adoption or introduction by Satsumon populations by at least a century, which may speak against the hypothesis that barley was introduced to northern Hokkaido by the more agrarian south.

The macrobotanical remains of barley are not enough evidence to argue for cultivation at the site, as opposed to the importing of grains from elsewhere. However, axe- and hoe-shaped bone tools found at nearby sites were likely farming implements and do support the possibility of local cultivation. In addition to low-level cultivation, the archaeobotanical data also suggests that wild plant management was conducted by the people of Rebun Island. The pollen record from Lake Kushu indicates significant local vegetation disturbance (i.e. deforestation) concurrent with the barley record at nearby Hamanaka 2. This reconstructed landscape patchiness may point to land clearance for small-scale crop cultivation. In view of the major role of marine food resources indicated by bone isotope studies, it seems likely that cultivated crops were used as supplementary food or for brewing beer. While future studies will clarify the role of barley in the economy in this region, it does seem clear that there was some kind of low-level crop production, which was complemented by wild foods. This rather recent example of incorporating domesticated crops into a foraging society adds to the ongoing debate about defining the middle ground landscape and further emphasises its great complexity. Moreover, it highlights the need for future studies to better understand what conditions control the adoption of agricultural practices by persistent non-agrarian societies, especially if such adoption seems to be economically inappropriate. A broad array of underlying factors should be considered, which may comprise climate, population pressure, social status, ritual practices, trade activities and so on.

So far, the archaeobotanical assemblages from the Hokkaido Okhotsk culture sites highlight the north-eastern limit of prehistoric barley dispersal. Seed morphological characteristics identified two different barley phenotypes, which were likely independently introduced to the Hokkaido Region. One highly compact type (naked barley) associated with the Okhotsk culture and a less compact type (likely hulled barley) that is evident in Early–Middle Satsumon culture sites. The much more comprehensive dataset presented in this paper supports earlier suggestions that the “Satsumon type” barley was likely propagated by the expansion of the Yayoi culture from south-western Japan towards north-eastern Japan, while the “Okhotsk type” spread from the continental RFE Region, across the Sea of Japan. Although Okhotsk populations may have obtained barley by exchange, there is growing data that suggest that they cultivated naked barley locally, which they introduced directly from their region of origin (i.e. the lower Amur River basin) via Sakhalin. To further verify this hypothesis, additional palaeobotanical studies on materials from archaeological sites in these areas are essential. Nevertheless, based on existing palaeobotanical evidence, we conclude that the Okhotsk culture represents one element of the long-term and spatially broader Holocene hunter–gatherer cultural complex (including also Jomon, Epi-Jomon, Satsumon, and Ainu cultures) of the Japanese archipelago, which may be placed into Smith’s [9] middle ground subsistence strategy. This middle ground domain may chronologically include the groups dating to the Neolithic–Iron Age interval (ca. 3300 cal yr BC–middle 1st millennium AD) and such cultures as Zaisanovskaia, Yankovskaia, and Krounovskaia of the coastal zone of today’s northern North Korea and the RFE, which share several subsistence traits with the Okhotsk culture.

## Supporting information

S1 Table Total counts of domesticated and wild seeds and floated litres from the Okhotsk culture layers of Hamanaka 2. (XLS)

S2 Table Morphological data of well-preserved carbonised naked barley seeds recovered from the Okhotsk culture layers of Hamanaka 2. (XLS)

S3 Table Morphological data of carbonised barley seeds from archaeological sites in the regions of Hokkaido, northern Tohoku, and the RFE collected from published and unpublished records. (XLS)
